# Pentoxifylline Neuroprotective Effects Are Possibly Related to Its Anti-Inflammatory and TNF-Alpha Inhibitory Properties, in the 6-OHDA Model of Parkinson's Disease

**DOI:** 10.1155/2015/108179

**Published:** 2015-09-29

**Authors:** Kelly Rose Tavares Neves, Hélio Vitoriano Nobre, Luzia Kalyne A. M. Leal, Geanne Matos de Andrade, Gerly Anne de Castro Brito, Glauce Socorro de Barros Viana

**Affiliations:** ^1^Faculty of Medicine of the Federal University of Ceará, Rua Coronel Nunes de Melo 1127, 60430-270 Fortaleza, CE, Brazil; ^2^Faculty of Medicine Estácio of Juazeiro do Norte, Avenida Tenente Raimundo Rocha 515, 63048-080 Juazeiro do Norte, CE, Brazil

## Abstract

Pentoxifylline (PTX) is a phosphodiesterase inhibitor with anti-TNF-alpha activity, associated with its anti-inflammatory action. Considering Parkinson's disease (PD) as a neuroinflammatory disorder, the objectives were to evaluate PTX neuroprotective properties, in a model of PD. Male Wistar rats, divided into sham-operated (SO), untreated 6-OHDA, and 6-OHDA treated with PTX (10, 25, and 50 mg/kg) groups, received a unilateral 6-OHDA injection, except the SO group administered with saline. Treatments started 24 h after surgery and continued for 15 days when the animals were submitted to apomorphine-induced rotations, open field, and forced swimming tests. At the next day, they were euthanized and their striata processed for neurochemical (DA and DOPAC determinations), histological, and immunohistochemical (Fluoro-Jade, TH, DAT, OX-42, TNF-alpha, COX-2, and iNOS) studies. PTX reversed the behavioral changes observed in the untreated 6-OHDA animals. Furthermore, PTX partially reversed the decrease in DA contents and improved neuronal viability. In addition, decreases in immunostaining for TH and dopamine transporter (DAT) were reversed. The untreated 6-OHDA group showed intense OX-42, TNF-alpha, COX-2, and iNOS immunoreactivities, which were attenuated by PTX. In conclusion, we demonstrated a neuroprotective effect of PTX, possibly related to its anti-inflammatory and antioxidant actions, indicating its potential as an adjunct treatment for PD.

## 1. Introduction

Pentoxifylline (PTX) is a nonselective phosphodiesterase inhibitor that decreases TNF-alpha gene transcription, affecting directly or indirectly multiple steps in the cytokine/chemokine pathways and exerting beneficial immunomodulatory effects in inflammatory conditions [1]. Evidence of increased levels of specific cytokines, including tumor necrosis factor-alpha (TNF-alpha), within nigrostriatal dopaminergic regions from Parkinson's disease (PD) patients points out that PD is also the result of immunological responses which promote increased synthesis and release of proinflammatory cytokines [2].

Cytokines, as TNF-alpha, are involved in the regulation of the central nervous system (CNS) and immune system interactions and are also important for the coordination of immune responses throughout the body. In the CNS, cytokines as well as chemokines function as neuromodulators and regulate neurodevelopment, neuroinflammation, and synaptic transmission. Furthermore, a recent work [3] showed that lowering TNF-alpha synthesis ameliorates neuronal cell loss and cognitive impairments in a model of traumatic brain injury in mice.

PD is a chronic neurodegenerative disease clinically characterized by bradykinesia, hypokinesia, rigidity, resting tremor, and postural instability. These motor manifestations are attributed to the degeneration and selective loss of dopaminergic neurons in the* substantia nigra pars compacta* (SNpc), leading to a dopamine (DA) deficiency in the striatum. Furthermore, the clinical diagnosis of PD rests on the identification of characteristics related to dopamine deficiency. However, nondopaminergic and nonmotor symptoms, including cognitive dysfunction and depression, which is one of the most common and persistent symptoms, are sometimes present at an earlier disease stage and, almost inevitably, emerge with the disease progression [4, 5].

Recently [6], we demonstrated that caffeine, a nonselective adenosine A_2A_ antagonist, as PTX, attenuates the striatal dopaminergic loss and decreases proinflammatory cytokines, as TNF-alpha and IL-1 beta, in the PD model of 6-OHDA lesion. Furthermore, PTX by inhibiting phosphodiesterase (PDE) activity decreases inflammation-related responses [7]. PDEs are responsible for the breakdown of cyclic nucleotides, as cAMP and cGMP, and their signaling has been associated with neuroplasticity and protection. Thus, inhibition of PDEs has become a target for the treatment of a wide array of disorders, including neurodegenerative ones [8]. In addition, PTX suppressed nitric oxide (NO) production and NO synthase at the mRNA level. NO suppression by PTX paralleled the increased cAMP levels, and these effects could be beneficial in NO-mediated inflammation [9].

Currently, no therapy is available that can slow down or halt the progression of PD or regenerate the affected brain regions [10]. PTX presents anti-inflammatory actions, and neuroinflammation is considered to be an important target for neuroprotection. Thus, the objectives of the present work were to support the neuroprotective effects of PTX, as previously shown by us, in a model of global brain ischemia, in rats [11]. Besides, TNF-alpha, a proinflammatory cytokine inhibited by PTX, is a link between neuroinflammation and excitotoxicity [12]. Our objectives were to use behavioral and neurochemical evaluations, as well as histological and immunohistochemical assays in rats subjected to the 6-OHDA model of hemiparkinsonism.

## 2. Materials and Methods

*Drugs and Reagents*. Pentoxifylline (Trental) was from Sanofi-Aventis (Suzano, São Paulo, Brazil); 6-hydroxydopamine, apomorphine, and HPLC standards were from Sigma-Aldrich (St. Louis, MO, USA); ketamine and xylazine were from Konig do Brasil (Santana de Parnaíba, São Paulo, Brazil). Antibodies for immunohistochemistry assays were from Santa Cruz Biotechnology (Dallas, TX, USA) or Merck-Millipore (Darmstadt, Germany). All other reagents were of analytical grade.

*Animals*. Male Wistar rats (200–250 g) were maintained at 24 ± 2°C temperature, in a 12 h dark/12 h light cycle, with standard food and water* ad libitum*. The study was submitted to the Ethical Committee for Animal Experimentation of the Faculty of Medicine of the Federal University of Ceará (Brazil) and was approved under the number 23/2010. All experiments followed the ethical principles established in the Guide for the Care and Use of Laboratory Animals, USA, 1986. 

*The 6-OHDA Model of PD and the Experimental Protocol*. The animals were anesthetized with the association of xylazine (10 mg/kg, i.p.) and ketamine (80 mg/kg, i.p.), submitted to shaving of the head superior region, and fixed to the stereotaxic frame by their ear canals. A longitudinal midline incision was made and tissues were separated for bregma visualization. Then, a thin hole was performed in the skull over the target area, and 1 *μ*L solution containing 6 *μ*g 6-OHDA was injected into two different points. The following coordinates were used: 1st point (AP, +0.5; ML, −2.5; DV, +5.0) and 2nd point (AP, −0.9; ML, −3.7; DV, +6.5). The syringe stayed in place for 5 min, to assure the solution diffusion, and then the incision was sutured. The sham-operated (SO) animals were subjected to all procedures, except that saline was injected into the two points. Afterwards, the animals returned to their cages for recovering. They were divided into the following groups: SO (treated by gavage with distilled water), 6-OHDA-lesioned (orally administered with distilled water), 6-OHDA-lesioned + PTX10, 6-OHDA-lesioned + PTX25, and 6-OHDA-lesioned + PTX50 (these last three groups were orally treated by gavage with PTX, at the doses of 10, 25, or 50 mg/kg). All treatments started 24 h after the surgical procedure and continued for 15 days, with drug volumes of 0.2 mL/100 g body weight. Following treatments and 1 h after the last drug administration, the animals were submitted to the behavioral tests. At the next day, they were euthanized (decapitation) and brain tissues removed for neurochemical, histological, and immunohistochemical studies.

### 2.1. Behavioral Testing and Neurochemical Determinations

*Apomorphine-Induced Rotations*. The contralateral rotations (opposite to the lesioned right side) induced by apomorphine (1 mg/kg, s.c.) were monitored for 1 h. The cause for this apomorphine-induced rotational behavior is related to the unbalance, in the nigrostriatal dopaminergic pathways, between the right (lesioned) and left (unlesioned) brain hemispheres. This asymmetric circling behavior after the apomorphine administration is a quantifiable motor deficit and an important paradigm in this model of PD.

*Open Field Test*. This test evaluates a stimulant or depressant drug activity and may also indicate an anxiolytic action. The arena was made of wood, whose dimensions were 50 cm × 50 cm × 30 cm (length, width, and height). The floor was divided into 4 quadrants of equal size. At the time of the experiment, the apparatus was illuminated by a red light and it was cleaned afterwards with a 70% alcohol solution to avoid odor interference in the test response. The following parameters were observed for 5 min: number of crossings with the four paws from one quadrant to another (this measures the locomotor spontaneous activity) and the number of rearing movements (stereotyped vertical exploratory movements). 

*Forced Swimming Test*. This test is based on the observation that when the animals are subjected to a stressful situation, with no possibility for escaping, they adopt a posture of immobility after an initial period of agitation. The reduction of this immobility time is suggestive of an antidepressant action. The animals were placed individually in a cylinder (40 cm in height and 23 cm in diameter), containing water up to 25 cm below the top. The immobility time was monitored for 5 min, after an initial 1 min adaptation period. 

*Neurochemical Determinations of DA and DOPAC by HPLC*. The striatal contents of DA and DOPAC were determined by HPLC. Homogenates were prepared in 10% HClO_4_ and centrifuged at 4°C (15,000 rpm, 15 min). The supernatants were filtered and 20 *µ*L injected into a column (Shim-Pak CLC-ODS, 25 cm) coupled to an electrochemical detector (model L-ECD-6A from Shimadzu, Japan) and a flow of 0.6 mL/min. For that, an electrochemical detector (model L-ECD-6A from Shimadzu, Japan) coupled to a column (Shim-Pak CLC-ODS, 25 cm) with a flux of 0.6 mL/min was employed. A mobile phase was prepared with monohydrated citric acid (150 mM), sodium octyl sulfate (67 mM), 2% tetrahydrofuran, and 4% acetonitrile, in deionized water. The mobile phase pH was adjusted to 3.0 with NAOH (10 mM). Monoamines were quantified by comparison with standards and processed the same manner as the samples. The results are expressed as ng/g tissue.

### 2.2. Histological and Immunohistochemical Analyses in Rat Striata

*Fluoro-Jade*. Fluoro-Jade is an anionic fluorescein derivative, useful for the histological staining of neurons undergoing degeneration. After paraffin removal (by immersion in xylol), sections (5 *μ*m) were mounted on slides surrounded by gelatin. The tissue was rehydrated by immersion in ethanol for 3 min, followed by immersions in 70 and 50% ethanol solutions and distilled water. The slices were placed into a 0.06% potassium permanganate solution, for 15 min, washed in distilled water, and transferred to a Fluoro-Jade solution where they stayed for 30 min (with gentle stirring). After staining, the slices were washed in distilled water (3 times, 2 min each time). The excess of water was discarded and the dry slices mounted in Fluoromount media and were examined with a fluorescence microscope.

*Immunohistochemistry Assays*. Brain striatal sections (5 *μ*m) were fixed in 10% buffered formol, for 24 h, followed by a 70% ethanol solution. The sections were embedded into paraffin wax for slices processing on appropriate glass slides. These were placed in the oven at 58°C, for 10 min, followed by deparaffinization in xylol, rehydration in alcohol at decreasing concentrations, and washing in distilled water and PBS (0.1 M sodium phosphate buffer, pH 7.2), for 10 min. The endogenous peroxidase was blocked with a 3% hydrogen peroxide solution, followed by incubation with the appropriate primary anti-antibody for tyrosine hydroxylase (TH), dopamine transporter (DAT), OX-42 (an antibody and marker for microglia), TNF-alpha, cyclooxygenase-2 (COX-2), or inducible nitric oxide synthase (iNOS) and diluted according to the manufacturers' instructions (Santa Cruz or Millipore, USA), for 2 h, at room temperature in a moist chamber. The glass slides were then washed with PBS (3 times, 5 min each) and incubated with the biotinylated secondary antibody, for 1 h, at room temperature. Then, they were washed again in PBS and incubated with streptavidin peroxidase, for 30 min, at room temperature. After another wash in PBS, they were incubated in 0.1% DAB solution (in 3% hydrogen peroxide). Finally, the glass slides were washed in distilled water and counterstained with Mayer's hematoxylin, washed in tap water, dehydrated in alcohol (at increasing concentrations), diaphanized in xylol, and mounted on Entellan for optic microscopy examination. The immunostaining intensity was quantified by the ImageJ software (National Institute of Health, USA) and the results were expressed as relatively optical.

### 2.3. Statistical Analyses

For statistical analyses, one-way ANOVA, followed by the Newman-Keuls as the* post hoc* test, was used for multiple comparisons. Whenever needed, paired or unpaired Student's* t*-test was used for comparisons between two means. Differences were considered significant at *p* < 0.05.

## 3. Results

### 3.1. Behavioral Testing

**Figure 1:**
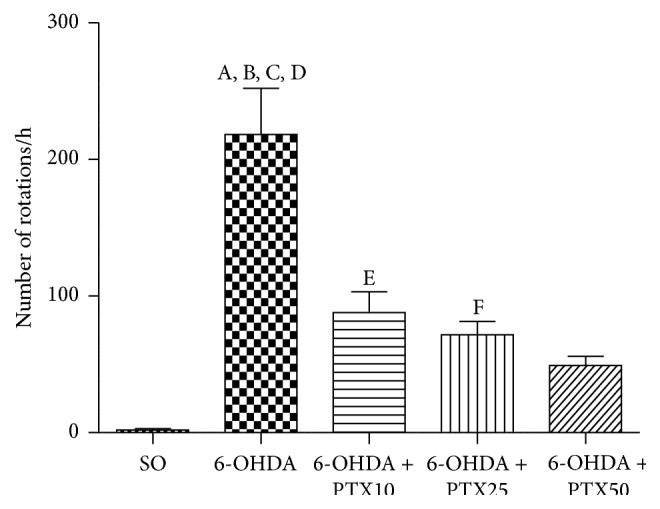
Pentoxifylline treatments (PTX: 10, 25, and 50 mg/kg, 15 days) decrease the apomorphine-induced rotational behavior in the 6-OHDA group, relatively to the untreated 6-OHDA group. The values are means ± SEM from 8 to 11 animals per group. A versus SO,* q* = 12.66 *p* < 0.0001; B versus 6-OHDA + PTX10,* q* = 7.211 *p* < 0.0001; C versus 6-OHDA + PTX25,* q* = 8.790 *p* < 0,0001; D versus 6-OHDA + PTX50,* q* = 10.14 *p* < 0.0001; E versus SO,* q* = 4.726 *p* < 0.001; F versus SO,* q* = 4.169 *p* < 0.01.  *F*(4,45) = 22.51, *p* < 0.0001 (one-way ANOVA and Newman-Keuls as the* post hoc* test).

*Apomorphine-Induced Rotations*. Fifteen days after the stereotaxic procedure and 1 h after the last drug administration, the animals were injected with apomorphine (1 mg/kg, s.c.) and observed for rotational behavior, for 1 h. The results showed almost no contralateral rotation in the SO group, while the untreated 6-OHDA group presented 218.3 ± 33.74 rotations/h. On the other hand, the number of contralateral rotations decreased by 60, 66, and 77% in 6-OHDA animals, after PTX treatments with 10, 25, and 50 mg/kg, respectively [*F*(4,45) = 22.51, *p* < 0.0001] (Figure 1). 

**Figure 2:**
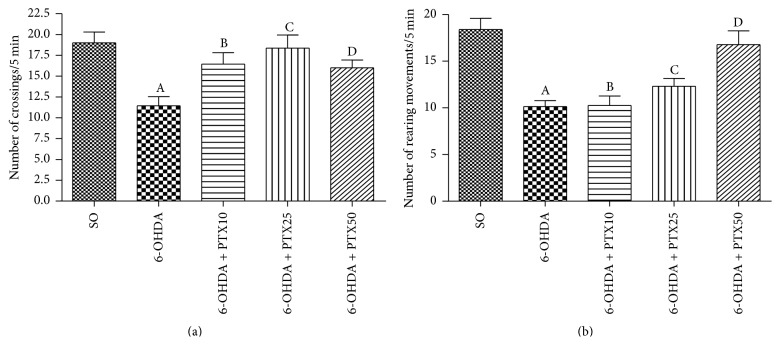
Pentoxifylline treatments (PTX: 10, 25, and 50 mg/kg, 15 days) reverse behavioral alterations in the 6-OHDA group, relatively to untreated 6-OHDA rats, as assessed by the open field test. The values are means ± SEM from 9 to 20 animals per group. (a) Number of crossings/5 min (locomotor activity): A versus SO, *p* < 0.0001; B versus 6-OHDA, *p* = 0.0110; C versus 6-OHDA, *p* = 0.0008; D versus 6-OHDA, *p* = 0.031. (b) Number of rearing movements/5 min (vertical exploratory activity): A versus SO, *p* < 0.0001; B versus SO, *p* = 0.0003; C versus SO, *p* = 0.0002; D versus 6-OHDA, *p* < 0.0001 (two-tailed Student's* t*-test).

*Locomotor Activity and Rearing Behavior*. While the untreated 6-OHDA groups showed around 32% decrease in locomotor activity, as related to the SO group, PTX treatments reversed this effect [*F*(4,73) = 6.532, *p* = 0.0002] bringing them to values not significantly different from those of the SO group (Figure 2(a)). Furthermore, the 37% decrease in rearing behavior [*F*(4,78) = 11.81, *p* < 0.0001], observed in the untreated 6-OHDA groups, was totally reversed after the higher PTX dose (Figure 2(b)).

**Figure 3:**
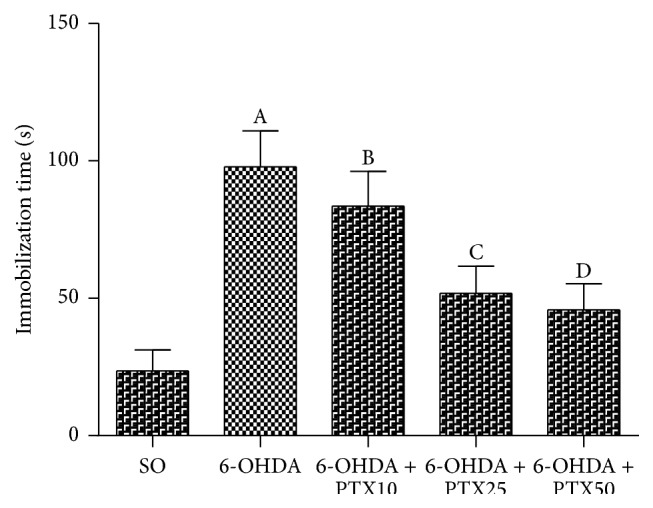
Pentoxifylline treatments (PTX: 10, 25, and 50 mg/kg, 15 days) reverse the decreased immobility time in the 6-OHDA group, relatively to the untreated 6-OHDA rats, in the forced swimming test. The values are means ± SEM from 8 to 9 animals per group. A and B versus SO; C and D versus 6-OHDA; *p* = 0.0234 to *p* < 0.05 (two-tailed Student's* t*-test).

*Immobility Time*. While the immobility time increased in the untreated 6-OHDA group, as related to the SO, this parameter decreased dose dependently in the 6-OHDA groups after PTX treatments with the two higher doses (significance levels: *p* = 0.0234 to *p* < 0.05). These data suggest that the drug, besides improving locomotor activity, ameliorates the depressive-like behavior observed in the untreated 6-OHDA group (Figure 3).

### 3.2. Neurochemical Assays

**Figure 4:**
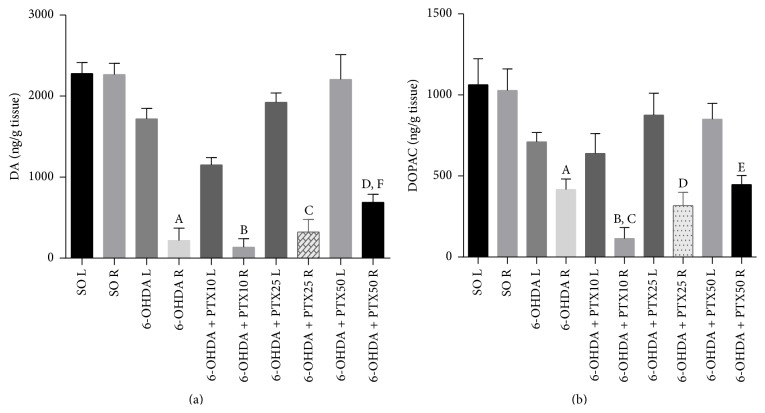
Pentoxifylline treatments (PTX: 10, 25, and 50 mg/kg, 15 days) reverse in part striatal dopamine (DA) and DOPAC depletions in the 6-OHDA group, relatively to the untreated 6-OHDA group. The values are means ± SEM from 6 to 10 animals per group. L and R correspond to the left (unlesioned) and right (lesioned) sides of the rat striata. (a) DA: A versus 6-OHDA L, *p* < 0.0001; B versus 6-OHDA + PTX10 L, *p* < 0.0001; C versus 6-OHDA + PTX25 L, *p* < 0.0001; D versus 6-OHDA + PTX50 L, *p* = 0.0011; F versus 6-OHDA R, *p* = 0.0003. (b) DOPAC: A versus 6-OHDA L, *p* = 0.0080; B versus 6-OHDA + PTX10 L, *p* = 0.0019; C versus 6-OHDA R, *p* = 0.0067; D versus 6-OHDA + PTX25 L, *p* = 0.0086; E versus 6-OHDA + PTX50, *p* = 0.0038 (two-tailed Student's* t*-test).

*DA and DOPAC Determinations*. The untreated 6-OHDA animals showed a 90% decrease of striatal DA contents in the lesioned right side, as related to their left side or to the right side of the SO group. While no significant effect was seen at lower doses, PTX treatments at the dose of 50 mg/kg showed DA concentrations higher than those of the untreated 6-OHDA group [*F*(9,65) = 39.41, *p* < 0.0001], indicative of a partial reversion of the DA depletion (Figure 4(a)). Interestingly, striatal DOPAC contents significantly [*F*(9,68) = 8.053, *p* < 0.0001] decreased in both sides of the untreated 6-OHDA group and this alteration was not reversed after PTX treatment (Figure 4(b)).

### 3.3. Histological Studies

**Figure 5:**
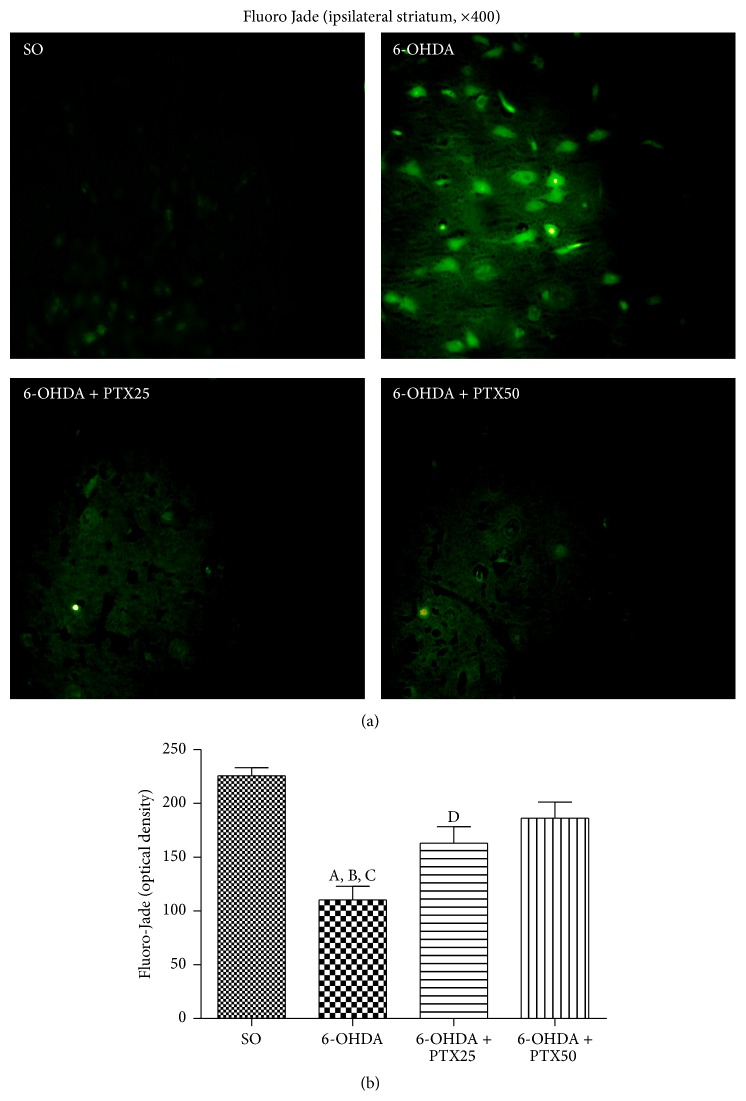
Representative photomicrographs showing that pentoxifylline treatments (PTX, 15 days) reverse in part the neuronal degeneration in the 6-OHDA group, relatively to the untreated 6-OHDA group, as evaluated by the Fluoro-Jade staining in the rat striatum. Groups: SO, untreated 6-OHDA, and 6-OHDA treated with PTX, at the doses of 25 and 50 mg/kg. Ipsilateral side = lesioned side. Magnification: 400x. A versus SO,* q* = 8.556 *p* < 0.0001; B versus 6-OHDA + PTX25,* q* = 4.164, *p* < 0.01; C versus 6-OHDA + PTX50,* q* = 5.636 *p* < 0.001; D versus SO,* q* = 4.475 *p* < 0.01 (one-way ANOVA and Newman-Keuls as the* post hoc* test).

*Fluoro-Jade*. A high number of fluorescent neurons were seen in the lesioned right striatum (ipsilateral side) of the untreated 6-OHDA group, indicative of neurons undergoing degeneration [13]. In this group, there was more than 50% neuron degeneration, as related to the SO group. The number of fluorescent neurons was much lower in the 6-OHDA group, after PTX treatments, mainly with the higher dose (50 mg/kg), which showed only 18% degeneration (Figure 5).

### 3.4. Immunohistochemical Studies

**Figure 6:**
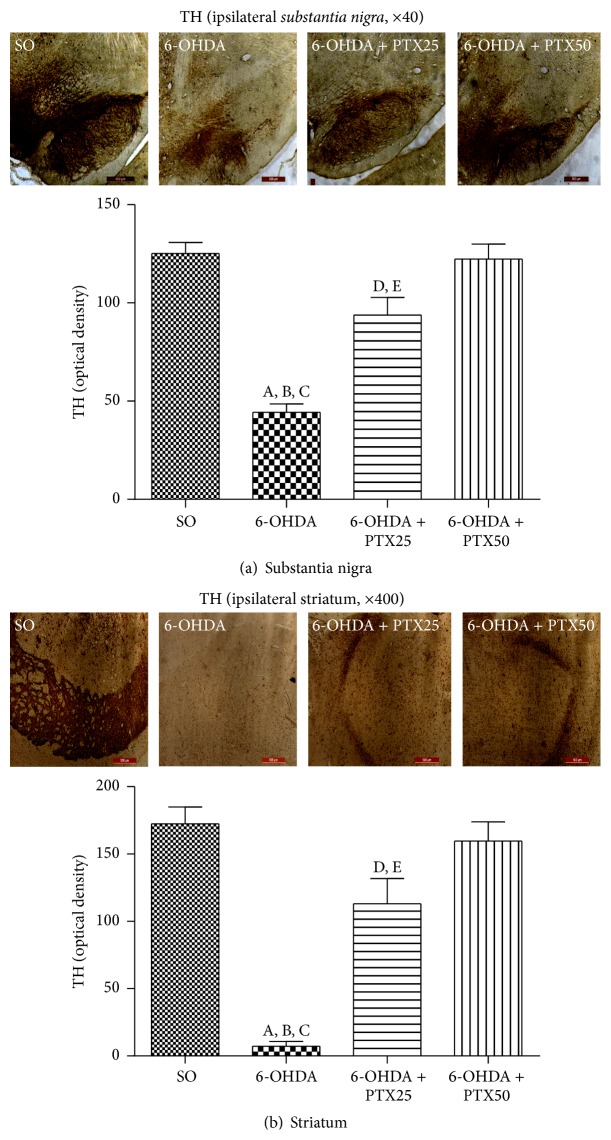
Representative photomicrographs showing that pentoxifylline treatments (PTX, 15 days) reverse the increased immunoreactivity for tyrosine hydroxylase (TH) in the 6-OHDA group, relatively to the* substantia nigra* and striatum of the untreated 6-OHDA group. Groups: SO (sham-operated, control), untreated 6-OHDA, and 6-OHDA treated with PTX, at the doses of 25 and 50 mg/kg. Ipsilateral = lesioned side. Magnifications: 40 and 400x.* Substantia nigra*: A versus SO,* q* = 11.76 *p* < 0.0001; B versus 6-OHDA + PTX25,* q* = 7.200 *p* < 0.0001; C versus 6-OHDA + PTX50,* q* = 11.35 *p* < 0.0001; D versus SO, *q* = 4.556 *p* < 0.01; E versus 6-OHDA + PTX25,* q* = 4.149 *p* < 0.001.* Striatum*: A versus SO,* q* = 12.26 *p* < 0.0001; B versus 6-OHDA + PTX25,* q* = 7.850 *p* < 0.0001; C versus 6-OHDA + PTX50,* q* = 11.31 *p* < 0.0001; D versus SO,* q* = 4.408 *p* < 0.01, E versus 6-OHDA + PTX25,* q* = 3.458 *p* < 0.01 (one-way ANOVA and Newman-Keuls as the* post hoc* test).

*Immunohistochemistry for Tyrosine Hydroxylase (TH)*. This is the rate-limiting step in the synthesis of brain catecholamines and it is considered a biomarker in experimental models of PD.  Figure 6(a) presents representative photomicrographs of the* substantia nigra* (ipsilateral side, 40x), showing much lower immunostainings in the untreated 6-OHDA, as related to the other groups (SO, PTX25, and PTX50).  Figure 6(b) shows a drastic decrease of 2.8-fold in cell immunoreactivity in the lesioned right striatum (ipsilateral side) of the untreated 6-OHDA group, as related to the SO group. The enzymatic immunoreactivity was unchanged in the striatum from the 6-OHDA group, after PTX treatments (25 and 50 mg/kg). 

**Figure 7:**
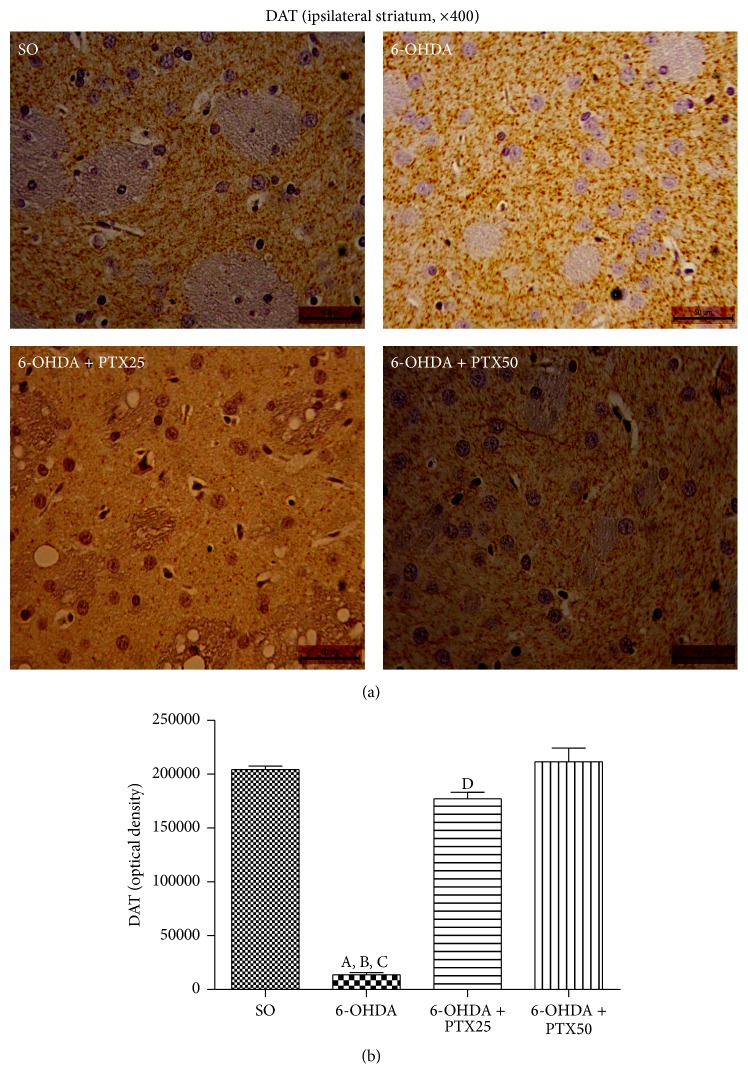
Representative photomicrographs showing that pentoxifylline treatments (PTX, 15 days) reverse the increased immunoreactivity for the dopamine transporter (DAT) in the 6-OHDA group, relatively to the striatum from the untreated 6-OHDA group. Groups: SO (sham-operated, control), untreated 6-OHDA, and 6-OHDA treated with PTX at the doses of 25 and 50 mg/kg. Ipsilateral = lesioned side, as compared to the unlesioned contralateral side. Magnification: 400x. A versus SO,* q* = 23.97 *p* < 0.0001; B versus 6-OHDA + PTX25,* q* = 20.54 *p* < 0.0001; C versus 6-OHDA + PTX50,* q* = 26.36 *p* < 0.0001; D versus SO,* q* = 3.247 *p* < 0.01; E versus 6-OHDA + PTX25,* q* = 4.323 *p* < 0.01 (one-way ANOVA and Newman-Keuls as the* post hoc* test).

*Immunohistochemistry for Dopamine Transporter (DAT)*. DAT may be the single most important determinant of extracellular dopamine concentrations. Due to the loss of dopaminergic terminals, this molecule may be reduced by 50 to 70% in PD and can also be considered a biomarker in this pathological condition [14].  Figure 7 shows differences in DAT immunoreactivities in the lesioned right striatum (ipsilateral side) from the untreated 6-OHDA group, with more than 90% less immunoreactivity as related to the SO group. The effect was in great part or totally reversed after PTX treatments (25 and 50 mg/kg, resp.). 

**Figure 8:**
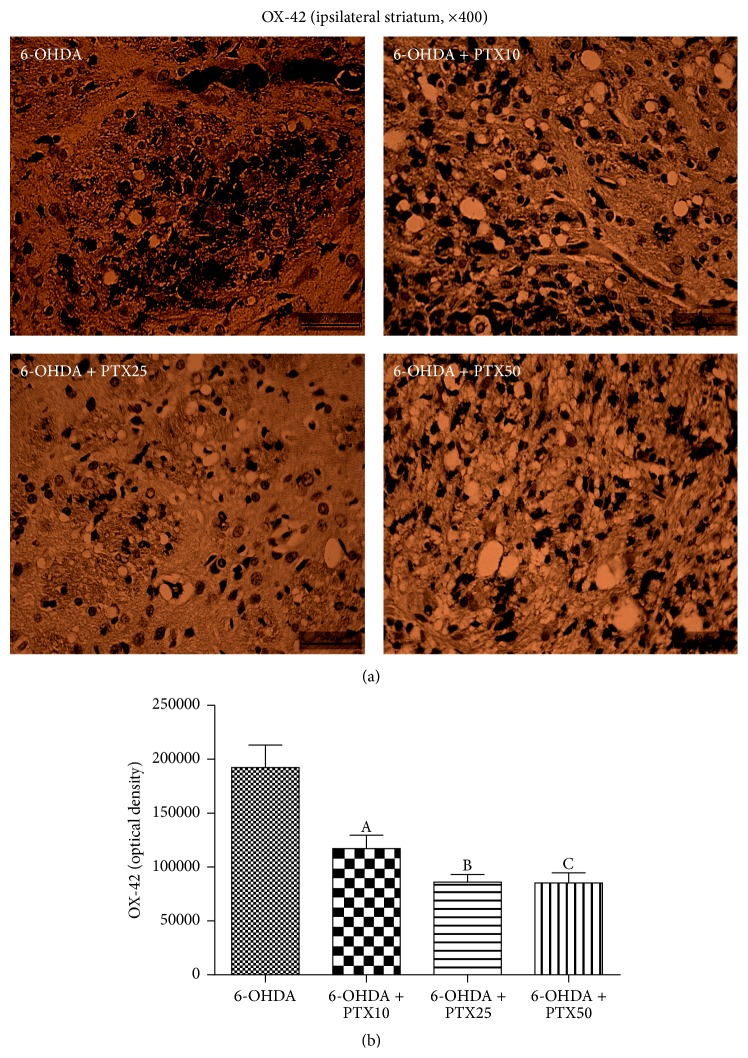
The lesioned ipsilateral striatum from the untreated 6-OHDA group shows high microglia immunoreactivities which significantly decreased after PTX treatments. A versus 6-OHDA + PTX10,* q* = 5.551 *p* < 0.001; B versus 6-OHDA + PTX25,* q* = 7.851 *p* < 0.001; C versus 6-OHDA + PTX50,* q* = 7.917 *p* < 0.001 (one-way ANOVA and Newman-Keuls as the* post hoc* test).

*Immunohistochemistry for OX-42*. Activated microglia in the* substantia nigra* of PD patients are an important factor in the disease neuroinflammation which is accompanied by the increased expression of proinflammatory cytokines [15]. While a great immunoreactivity for OX-42 (a microglial marker) was seen in the lesioned striatum of the untreated 6-OHDA group, this decreased by 39 and 55% after treatments with PTX, at the doses of 25 and 50 mg/kg, respectively (Figure 8). 

**Figure 9:**
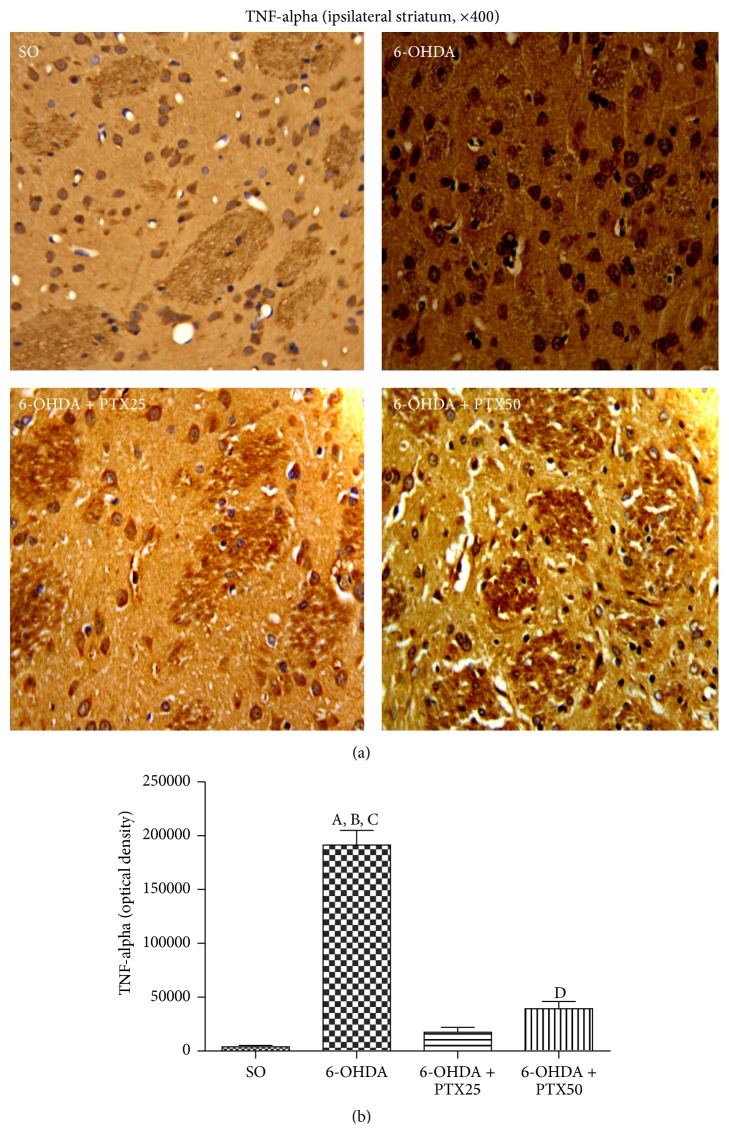
Representative photomicrographs showing that pentoxifylline treatments (PTX, 15 days) reverse the increased immunoreactivity for TNF-alpha in the 6-OHDA group, relatively to the ipsilateral striatum from the untreated 6-OHDA rats. Groups: SO (sham-operated, control), untreated 6-OHDA, and 6-OHDA treated with PTX, at the doses of 25 and 50 mg/kg (400x magnification). A versus SO,* q* = 23.85 *p* < 0.0001; B versus 6-OHDA + PTX25,* q* = 22.11 *p* < 0.0001; C versus 6-OHDA + PTX50,* q* = 19.34 *p* < 0.0001; D versus SO,* q* = 4.51 *p* < 0.01 (one-way ANOVA and Newman-Keuls as the* post hoc* test).

*Immunohistochemistry for TNF-Alpha*. Several studies have reported a relation between increased proinflammatory cytokines, as TNF-alpha, and neurodegenerative diseases, including PD [16]. We showed (Figure 9) a 50-fold increase in cell immunoreactivity for TNF-alpha, in the ipsilateral striatum of the untreated 6-OHDA group, as related to the SO group. On the other hand, the immunoreactivity decreased towards the SO pattern, in the 6-OHDA groups after PTX treatments (25 and 50 mg/kg). Surprisingly, while the decrease was only around 4.6-fold after 25 mg/kg PTX, it was around 10-fold after the treatment with the higher dose (50 mg/kg). 

**Figure 10:**
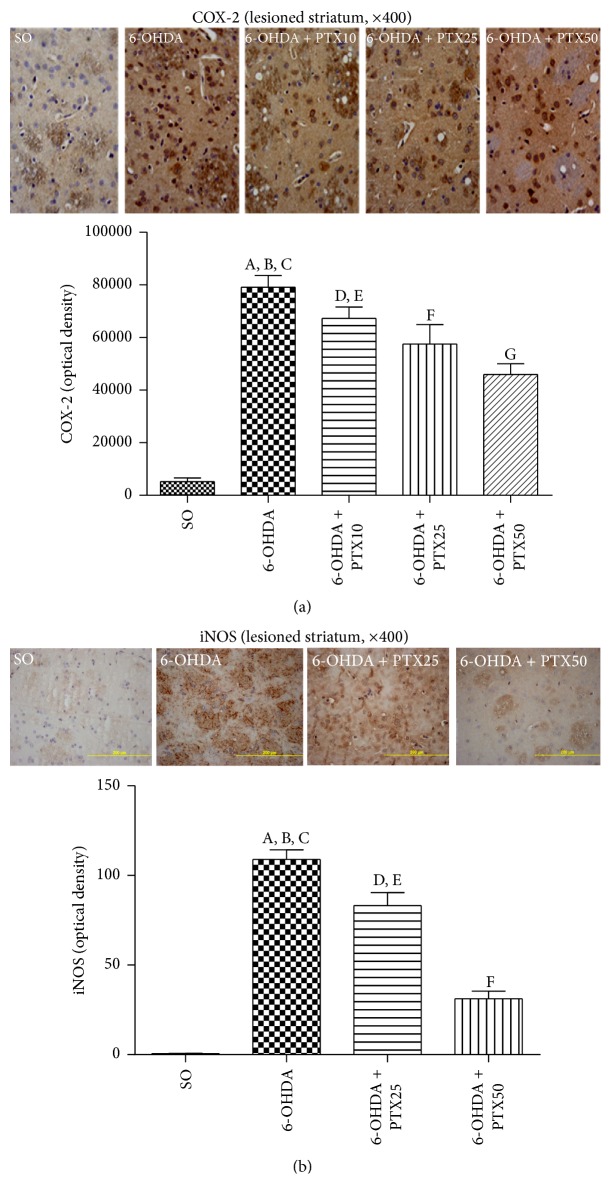
Representative photomicrographs from striata showing that PTX treatments decrease the immunostaining for COX-2 of 6-OHDA-lesioned rats. A similar profile was seen for iNOS immunostaining in the ipsilateral striatum of 6-OHDA-lesioned rats, after PTX treatments. COX-2: A versus SO,* q* = 9.529 *p* < 0.0001; B versus 6-OHDA + PTX25,* q* = 4.319 *p* < 0.001; C versus 6-OHDA + PTX50,* q* = 5.192 *p* < 0.001; D versus SO,* q* = 7.932 *p* < 0.0001; E versus SO,* q* = 4.766 *p* < 0.001; F versus SO,* q* = 3.894 *p* < 0.01; G versus 6-OHDA + PTX10,* q* = 3.669 *p* < 0.01. iNOS: A versus SO,* q* = 21.92 *p* < 0.0001; B versus 6-OHDA + PTX25,* q* = 5.208 *p* < 0.001; C versus 6-OHDA + PTX50,* q* = 15.73 *p* < 0.0001; D versus SO,* q* = 16.71 *p* < 0.0001; E versus SO,* q* = 6.193 *p* < 0.001; F versus 6-OHDA + PTX50,* q* = 10.52 *p* < 0.001 (one-way ANOVA and Newman-Keuls as the* post hoc* test).

*Immunohistochemistry for COX-2 and iNOS*. The neuroinflammatory reaction observed in PD brains is manifested not only by elevated cytokine levels but also by upregulation of COX-2 and iNOS [17]. We showed that while intense immunostainings for COX-2 (Figure 10(a)) and iNOS (Figure 10(b)) were demonstrated in the striatum from untreated 6-OHDA rats, these decreased highly after treatments with PTX, at the doses of 25 and 50 mg/kg. As a matter of fact, the increase in COX-2 immunoreactivity was around 8.5-fold in the ipsilateral striatum of the untreated 6-OHDA group, as related to the SO group, while it was around 4.9- and 4.2-fold after PTX treatments with 25 and 50 mg/kg, respectively. The increases were even higher (191-fold) for iNOS immunoreactivities in the striatum of the untreated 6-OHDA group, as related to the SO group, and decreased to lower values after PTX treatments (55-fold with the higher dose).

## 4. Discussion

The pathologic hallmark of PD is the loss of dopaminergic innervation in the striatum and the subsequent degeneration of dopaminergic neurons from SNpc, even though the degenerative process extends beyond that [18]. The pharmacologic treatment of PD can be divided into neuroprotective and symptomatic therapies. However, nearly all available treatments are symptomatic in nature and do not appear to slow or reverse the natural course of the disease. Since there are limited options for PD treatment, neuroprotective agents are currently being tested as means to slow down the disease progression [19].

In the present work, we studied the possible neuroprotective action of pentoxifylline. Our results showed behavioral alterations, in untreated 6-OHDA-lesioned rats, manifested by increased rotations after the apomorphine injection. This circling behavior is a consequence of neuron dopaminergic loss and was attenuated by PTX treatments, suggesting a neuroprotective effect. Furthermore, PTX treatments partly reversed the decreased locomotor activity, as related to untreated lesioned animals. The motor manifestations of PD are attributable to the degeneration of dopaminergic neurons within SNpc, resulting in DA depletion and derangements of neuronal circuits in the target regions of these neurons [20].

In addition, PTX also reversed the increased immobility time observed in the lesioned animals, suggesting an antidepressant-like effect. Others [21] showed that PTX reversed the depressive behavior, demonstrated in a model of myocardial infarction in rats. Depression is one of the most common and persistent nonmotor syndromes affecting roughly 40% of PD patients [5, 22]. Clinically significant symptoms of depression often emerge, before the motor symptoms, persisting throughout the course of the disease [23]. The striatal distribution of adenosine A_2A_ receptors and the antagonistic molecular and behavioral interactions between adenosine and dopamine receptors provide a strong basis for the clinical observation that A_2A_ receptor antagonists enhance motor activity in PD [24]. Interestingly, a clinical study showed that TNF-alpha antagonism may improve depressive symptoms in patients with high baseline inflammatory biomarkers [25].

A striking feature of PD is the preferential loss of DA-producing neurons in the midbrain. It has been proposed that the defective sequestration of DA into vesicles, leading to the generation of reactive oxygen species in the cytoplasm, is a key event in the demise of these neurons in PD and might represent a common pathway, underlying both genetic and sporadic forms of the disease [26]. Thus, despite representing only a symptomatic approach, most of the current therapy for PD focuses on the improvement of the brain DA contents. We showed that PTX, at the higher dose, attenuates the decreased striatal DA levels seen in lesioned animals.

Synaptic degeneration and death of neurons are defining features of PD, while DA-producing neurons in the* substantia nigra striatum* selectively degenerate [27]. Our results showed a higher fluorescence, indicative of neuron degeneration as demonstrated by the Fluoro-Jade staining in the striatum of the lesioned group, and this feature was greatly attenuated after PTX treatments.

Tyrosine hydroxylase (TH) is the rate-limiting enzyme in brain catecholamine biosynthesis and catalyzes the formation of L-DOPA, the rate-limiting step in the biosynthesis of DA. Thus, PD can be considered as a TH-deficiency syndrome in the striatum [28, 29]. In the present work, the striatal TH immunoreactivity was highly reduced in the ipsilateral side of the lesioned group, and this effect was reversed by PTX dose dependently. Similar results were demonstrated in the immunohistochemical data for the striatal dopamine transporter (DAT).

An important functional role of the dopamine transporter (DAT) is to maintain synaptic DA levels relatively constant and to preserve DA in nerve terminals. A decrease in DAT, despite potentially serving as a compensatory mechanism in early disease, may ultimately result in increased DA turnover and higher oscillations in synaptic DA concentrations, thereby possibly predisposing towards the occurrence of motor complications, as the disease progresses [30]. Thus, PTX effects on these two PD biomarkers, TH and DAT, make this drug potentially useful for PD treatment.

It is largely accepted that there is extensive communication between the immune system and the CNS and that acute or chronic neuron degeneration is associated with microglia activation and release of proinflammatory cytokines [31]. Furthermore, chronic neuroinflammation mediated by microglia plays an essential role in the death of dopaminergic neurons in PD [32–36]. We showed that microglia cells become highly reactive in the ipsilateral striatum of the untreated 6-OHDA animals. On the other hand, a much lower reactivity was observed after PTX treatments, indicating a neuroprotective property. A_2A_ receptor antagonists are potential neuroprotective drugs and the attenuation of microglial NO production could contribute to this neuroprotection [37, 38].

The underlying chronic inflammatory state, evident in PD, strongly suggests a role for neuroinflammation in dopaminergic cell death [39]. Thus, studies by Sawada et al., 2006 [40], reported a marked increase of cytokine levels, in the brain and cerebrospinal fluid of PD patients, and a higher density of glial cells that express TNF-alpha, IL-1 beta, and other cytokines in SNpc of PD patients, as compared to age-matched controls. We showed that while the untreated lesioned animals presented a high immunoreactivity for TNF-alpha, PTX decreased the number of immunopositive cells in the striatum. Evidences [16] indicate that PTX has the potential to inhibit proinflammatory and proapoptotic pathways, via the suppression of TNF-alpha and a caspase-dependent pathway in neuronal PC12 cells, suggesting its protective effects against inflammation-mediated neurodegeneration. Interestingly, recent data indicate that nonmotor features of PD, including depression, are associated with higher CSF levels of inflammatory markers [41].

Apart from the massive loss of dopaminergic neurons, PD brains also show a glial reactivity and neuroinflammation that, besides elevated cytokine levels, are manifested by upregulation of inflammation-associated factors, such as COX-2 and iNOS [17, 42]. In the present study, we clearly showed that PTX treatments of 6-OHDA-lesioned rats decrease immunostainings for COX-2 and iNOS. These data, together with PTX inhibition of TNF-alpha immunostaining, strongly favor the point that PTX neuroprotective action is related to its anti-inflammatory activity.

**Figure 11:**
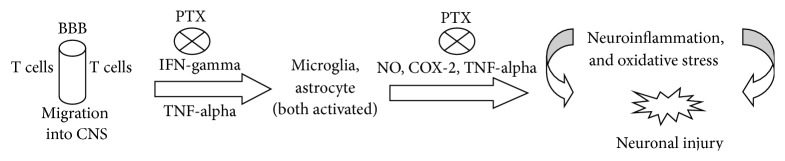
PTX would act by inhibiting steps involved in neuroinflammation and oxidative stress present in PD. Although astrocytes are able to produce TNF-alpha, among other factors, microglia are the major source of this cytokine, during neuroinflammation. Furthermore, INF-gamma is a potent inducer of TNF-alpha gene expression in microglia [12]. BBB = blood brain barrier.

PTX, besides being a phosphodiesterase inhibitor, increases cAMP levels and decreases TNF-alpha, presenting anti-inflammatory and antioxidant properties, as already shown by us [43] and others [7, 9]. The anti-inflammatory action of PTX is probably related to its ability to suppress oxygen radical production, scavenge reactive oxygen species [44], and blockade extracellular regulated kinase (ERK) phosphorylation and TNF-alpha production [45].  Figure 11 shows sites of possible PTX interventions. A common link in neurodegenerative diseases, including PD, is the presence of neuroinflammatory processes, justifying the importance of anti-inflammatory properties in a potentially neuroprotective drug. Recently [46], we also demonstrated the valproic acid neuroprotection in a PD model. This effect is possibly related to the drug anti-inflammatory action which is, at least partly, the result of its histone deacetylase (HDAC) inhibition. The enzyme HDAC display multiple roles in signaling pathways, and pharmacological modulators of this enzyme possess potent anti-inflammatory and protective effects in several neurological conditions, including neurodegenerative diseases. However, in the particular case of PTX, we believe that the drug anti-inflammatory action is mainly due to its anti-TNF-alpha effect, associated with iNOS and COX-2 inhibitions.

In conclusion, our data demonstrated that PTX exerts a neuroprotective activity in the 6-OHDA model of PD in rats. PTX is also A_2A_ receptor antagonist and these receptors, besides being abundantly expressed within the basal ganglia, are targets to modify abnormal striatal signaling associated with PD [47]. Evidences indicate that A_2A_ receptors signaling pathways mediate the anti-inflammatory effects of PTX [1]. Furthermore, PTX, by increasing the levels of DA, TH, and DAT and decreasing TNF-alpha, appears as a potential candidate to be included in clinical trials, alone or associated with other anti-inflammatory drugs, for the treatment of neurodegenerative pathologies as Parkinson's disease.
